# Genotype–Phenotype Relationship in Hereditary Hemorrhagic Telangiectasia: Quality of Life and Cardiovascular Risk Evaluation

**DOI:** 10.3390/jcm14134409

**Published:** 2025-06-20

**Authors:** Adrián Viteri-Noël, José Luis Patier, Nuria Bara-Ledesma, Andrés González-García, Martin Fabregate, Patricia Fernández-San Jose, Mónica López-Rodriguez, Luis Manzano, Vicente Gómez del Olmo

**Affiliations:** 1 Internal Medicine Department, Hospital Universitario Ramón y Cajal, Instituto Ramón y Cajal Investigación Sanitario, Colmenar Viejo, 28034 Madrid, Spain; patier43@hotmail.com (J.L.P.); nuriabara.mi@gmail.com (N.B.-L.); andres_gonzalez_garcia@hotmail.com (A.G.-G.); martin.fabregate.mi@gmail.com (M.F.); loprodmo@gmail.com (M.L.-R.); luis.manzano@uah.es (L.M.); vgomezdelolmo@hotmail.com (V.G.d.O.); 2 Faculty of Medicine and Health Sciences, Universidad de Alcalá (UAH), Av. de Madrid, 28871 Alcalá de Henares, Spain; 3 Department of Genetics, Hospital Universitario Ramón y Cajal, Instituto Ramón y Cajal Investigación Sanitario, Colmenar Viejo, 28034 Madrid, Spain; patriciafernandezsanjose@gmail.com; 4 Centro de Investigación Biomédica en Red de Enfermedades Raras, Instituto de Salud Carlos III (CB06/07/0048; CIBERER-ISCIII), 28029 Madrid, Spain

**Keywords:** HHT, Rendu–Osler–Weber syndrome, genotype, phenotype, epistaxis

## Abstract

Hereditary hemorrhagic telangiectasia (HHT) is an autosomal dominant vascular disorder caused by pathogenic variants in *ENG* (HHT1) and *ACVRL1* (HHT2), with distinct phenotypic expressions. **Background/Objectives**: This study investigates the genotype–phenotype correlations, including comparing the quality of life by phenotype, conducting a cardiovascular risk assessment, and evaluating the impact of mutation type on its clinical manifestations and prognosis. **Methods**: A cross-sectional study was conducted on 85 HHT patients, stratified into HHT1 (*ENG*, n = 43) and HHT2 (*ACVRL1*, n = 42). Their clinical and biochemical parameters, arteriovenous malformations (AVMs), epistaxis severity, quality of life, and cardiovascular risk were assessed. Genetic variants were classified as truncating or non-truncating. The statistical analyses included logistic regression and survival analysis. **Results**: The onset of epistaxis occurred earlier in HHT1 (log-rank *p* = 0.011), whereas its severity (*p* = 0.006) and iron deficiency were greater in HHT2 (*p* = 0.043). Pulmonary AVMs were significantly more frequent in HHT1 (58.1% vs. 9.5%, *p* < 0.01), contributing to a potential decrease in survival, despite the greater hemorrhagic burden in HHT2. Truncating mutations were independently associated with anemia (*p* < 0.05). Cardiovascular risk (measured using the SCORE2 scale) was low to moderate, and quality of life (measured using the EQ-5D-5L scale) was most impaired in patients with severe epistaxis (*p* = 0.031) or anemia (*p* = 0.026). Truncating mutations influence the severity of anemia independently of genotype. Limitations: The principal limitations include the small sample size and the bias generated by this being a paper based on another prospective study with a methodology designed for different objectives. **Conclusions**: These findings underscore the need for personalized management strategies based on genotype and mutation type. Further prospective studies are warranted to validate these associations and optimize the risk stratification in HHT.

## 1. Introduction

Hereditary hemorrhagic telangiectasia (HHT), also referred to as Rendu–Osler–Weber syndrome, is a rare disorder characterized by multisystemic vascular dysplasia [1].

The clinical manifestations of HHT are highly variable and can differ significantly even among individuals within the same family. According to recent estimates, its prevalence is approximately 1 in 5000 people [2]. However, certain geographic regions exhibit higher frequencies, including the Dutch Antilles, the island of Funen (Denmark), the French region of Ain, Vermont (USA), Newcastle (the United Kingdom), and Las Palmas de Gran Canaria (Spain) [3].

In about 90% of cases, HHT results from a heterozygous mutation in either the ENG (endoglin) gene or the *ACVRL1* (activin-like receptor kinase 1, also known as ALK1) gene, following an autosomal dominant inheritance pattern. Additionally, mutations in SMAD4 have been identified in approximately 2% of patients, leading to a combined condition known as PJ-HHT syndrome, which is characterized by juvenile polyps and anemia. Mutations in ENG, located on chromosome 9 (9q3.3-q3.4), cause HHT type 1 (HHT1), whereas mutations in *ACVRL1*, located on chromosome 12 (12q13), result in HHT type 2 (HHT2) [4].

Common symptoms include epistaxis and extramucosal telangiectasias. Recurrent spontaneous epistaxis (nosebleeds) occurs in about 50% of patients by the age of 20 and in up to 90% by the age of 40. They are the main cause of functional impairment in these patients since they lead to iron deficiency and anemia [4]. The severity of this symptom is measured using the ESS (Epistaxis Severity Score) scale, which includes six questions on the frequency and intensity of bleeding and the need for medical attention [5]. Telangiectasias are small vascular malformations affecting the arterioles and venules. They commonly occur on the lips, tongue, face, fingers, and gastrointestinal tract. Due to their fragility, they are prone to bleeding and can significantly impact patients’ quality of life [6].

AVMs are a key feature of HHT, affecting multiple organs with varying severity. Pulmonary AVMs (common in HHT1) can cause hypoxemia and embolic risks. Hepatic AVMs (linked to *ACVRL1* mutations) may lead to heart failure and portal hypertension. Gastrointestinal AVMs cause chronic bleeding, especially in ENG mutation carriers. Cerebral AVMs, though less frequent, carry a risk of intracranial hemorrhage [4,7,8,9]. At present, there are already studies showing the correlation between the phenotype and genotype of different AVMs in patients with HHT. However, this work expands the knowledge on new genotypic–phenotypic correlations, including laboratory data, quality of life, and a cardiovascular risk assessment.

## 2. Materials and Methods

This study was conducted as an observational, cross-sectional analysis of 85 patients diagnosed with hereditary hemorrhagic telangiectasia (HHT). This study is nested within another prospective project [NCT05550376], so its methodology was adapted to this one. The selected patients attended a specific HHT consultation on a scheduled basis as standard clinical practice. Participants were classified into two groups based on genetic subtype: HHT1 (*ENG* mutation, n = 43) and HHT2 (*ACVRL1* mutation, n = 42).

### 2.1. Data Collection

The patients included were aged 18–75 years, had a confirmed HHT diagnosis based on the Curaçao criteria or genetic testing, were clinically stable (with an Epistaxis Severity Score [ESS] < 7), and provided informed consent. The exclusion criteria included recent major cardiovascular events, severe intercurrent illnesses, recent major surgery, continuous use of immunosuppressive or chemotherapy agents, substance abuse, pregnancy, or any condition preventing adherence to the study protocols [NCT05550376].

Demographic, clinical, physical examination, and biochemical variables were recorded at the beginning of the study. Against this background, a total of 85 patients were selected, 43 patients with an *ENG* mutation and 42 patients with an *ACVRL1* mutation. The sample size was predetermined by the study mentioned above.

### 2.2. The Mutational Analysis

All patients underwent mutation identification, through Sanger sequencing for older diagnoses and Next-Generation Sequencing (NGS) for more recent cases. The distribution of the mutation types in the 85 selected patients was analyzed. A genetic map was drawn up with the different mutations found in the patients from the cohort analyzed.

### 2.3. Variables and Definitions

- Epistaxis Severity Score (ESS): This was used to assess the frequency and intensity of epistaxis episodes (0–10). ESSs were then stratified according to severity. This created categories consisting of no epistaxis (an ESS < 1), mild epistaxis (an ESS between 1 and 4), and moderate epistaxis (an ESS ≥ 4) [5]
- Arteriovenous malformations (AVMs): These were evaluated based on location (pulmonary, hepatic, cerebral, spinal, or digestive) and age at diagnosis.
- Anemia: Hemoglobin and iron levels were measured.
- Renal and hepatic function: Serum creatinine; glomerular filtration rates (GFRs) calculated using the MDRD and CKD-EPI; liver enzymes (AST, ALT, GGT); and lipid profiles were analyzed.
- Cardiovascular risk assessment: The SCORE2 model was used to estimate 10-year cardiovascular risk, stratifying patients into low-, intermediate-, and high-risk categories.
- The quality of life assessment was carried out using the EQ-5D-5L survey. The investigators personally asked the patients the questions during the consultation and completed the surveys themselves. This scale is based on 5 main components: ‘mobility’, ‘self-care’, ‘activities of daily living’, ‘pain/discomfort’, and ‘anxiety/depression’.
- Genetic analysis: The classification of pathogenic variants included missense, nonsense, deletion, insertion, duplication, splicing, and other rare mutations. To assess the impact of mutation type on phenotype expression, genetic variants were categorized into truncating mutations (including nonsense and deletion/insertion mutations) and non-truncating mutations (including missense and splicing mutations).

### 2.4. The Statistical Analysis

Variables were described using the mean/median and the standard deviation/interquartile range or frequency and percentage. Normality was tested using the Shapiro–Wilk test and a graphical representation. Group comparisons were performed using

- Student’s *t*-test or the Mann–Whitney U test for quantitative variables, depending on the distribution.
- The paired Student’s *t*-test or the Wilcoxon test for repeated measures.
- The Chi-square test for associations between categorical variables.
- Pearson’s correlation coefficient for linear associations.
- Survival analyses were performed using the Kaplan–Meier method for estimation of the survival curves, and the log-rank test was used to compare the age of onset of epistaxis between genotypes, assessing the differences in the epistaxis-free survival curves.
- A logistic regression analysis was used to assess the association of truncated variants with anemia, adjusting for age, sex, and genotype. A *p*-value < 0.05 was considered statistically significant. The statistical analyses were performed using SPSS version 22 stadistical software.

## 3. Results

### 3.1. The Baseline Characteristics and Genotype Implications

A total of 85 patients with a clinically stable diagnosis of HHT were included, with 43 (50.5%) having *ENG* gene involvement and 42 (49.5%) having *ACVRL1* gene involvement. The mean age of the population was 48.6 ± 16.0 years, and 50 (58.8%) were female. A total of 24 (28.2%) patients met all four Curaçao criteria, while 53 (62.4%) met three criteria, leaving 8 (9.4%) with a probable diagnosis but confirmed through genetic testing (Table 1).

### 3.2. Epistaxis

**Figure 1:**
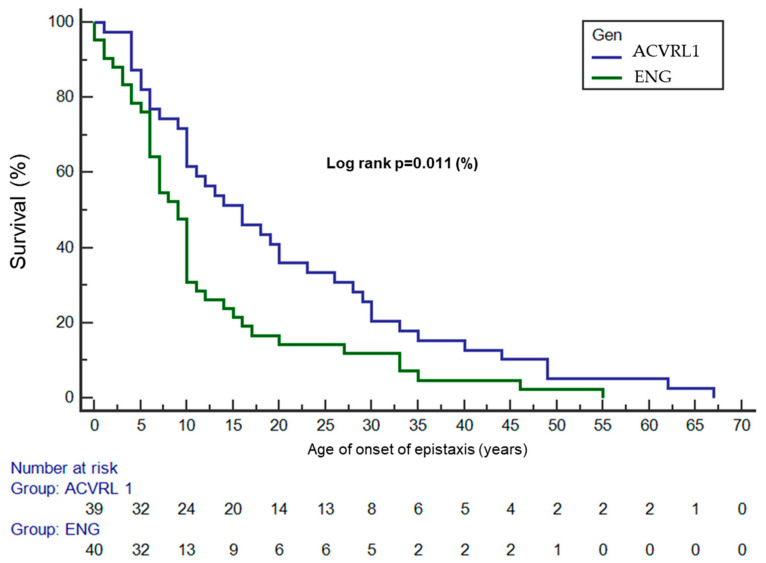
Age of onset of epistaxis.

Epistaxis was the most common symptom, affecting 76 (92.7%) patients, with a mean severity score (ESS) of 2.49 ± 1.79. No significant differences were found between the two genotypes in terms of their overall ESSs, although the patients with *ACVRL1* involvement had longer epistaxis episodes, with 21 of the patients in the *ENG* group experiencing episodes of less than one minute compared to only 7 in the *ACVRL1* group (*p* = 0.006). The patients with *ENG* mutations experienced epistaxis at an earlier age, as illustrated in Figure 1, with a significant difference in the epistaxis-free survival curves (log-rank *p* = 0.011).

Despite this, the severity of epistaxis appeared higher in the *ACVRL1* patients (Table 2). The duration of epistaxis was longer in the HHT2 patients (*p* = 0.006).

### 3.3. Telangiectasias

Telangiectasias were observed in 72 patients (84.7%), with no statistically significant differences between genotypes.

### 3.4. Vascular Malformations

A total of 44 (51.8%) patients presented with vascular malformations (VMs) in their internal organs. The presence of VMs was significantly higher in the ENG patients, with 27 (62.8%) presenting with VMs compared to 17 (40.5%) in the *ACVRL1* group (OR = 2.48, *p* = 0.040). Pulmonary arteriovenous malformations (AVMs) were the most frequent, observed in 29 (34.1%) patients, with a significantly higher presence in the *ENG* group (25 [58.1%] vs. 4 [9.5%], *p* < 0.01). The median age at diagnosis of pulmonary AVMs was 39.0 years [IQR: 24.0], with no significant differences between groups. Central nervous system VMs were present in 12 (14.1%) patients, with a tendency towards a higher prevalence in the ENG group (9 [20.9%] vs. 3 [7.1%], *p* = 0.068). The median age at diagnosis of CNS VMs was 28.0 years [IQR: 10.0], showing an earlier onset compared to that for other VMs. Hepatic VMs were observed in 15 (17.6%) patients, with a higher prevalence in the *ACVRL1* group (10 [23.8%] vs. 5 [11.6%], *p* = 0.141). The median age at diagnosis of hepatic VMs was 48.5 years [IQR: 19.5], with no differences between groups. Digestive VMs were identified in 10 (11.8%) patients, with no significant differences between genotypes and a median age at diagnosis of 57.5 years [IQR: 19.3]. In summary, CNS VMs were diagnosed earlier (median: 28 years), followed by pulmonary (39 years), hepatic (48 years), and digestive (57 years) VMs (Table 3).

### 3.5. Family History and Mortality

A total of 24 (28.2%) patients had relatives who had died due to HHT-related complications, with a trend towards higher mortality in *ENG* carriers (16 [18.8%] vs. 8 [9.4%], OR = 2.519, *p* = 0.063). The most common causes of death included severe anemia, massive epistaxis, pulmonary hemorrhage, pulmonary hypertension, and ischemic coronary artery disease, which together accounted for 11 (12.9%) deaths. Digestive bleeding was responsible for 10 (11.8%) deaths, while hemorrhagic stroke accounted for 7 (8.2%), with a trend towards a higher frequency in the *ENG* group (6 [14.0%] vs. 1 [2.4%], OR = 6.649, *p* = 0.052). The results are presented in Table 1.

### 3.6. Biochemical Differences

Iron metabolism differed between genotypes, with significantly higher serum iron levels in the *ENG* group (92.3 ± 72.7 mg/dL) compared to those in the *ACVRL1* group (66.8 ± 30.5 mg/dL, *p* = 0.043). Transferrin saturation was also higher in the *ENG* group (28.7% ± 26.2) than in the *ACVRL1* group (19.7% ± 9.2, *p* = 0.042). The ferritin levels were lower in the *ENG* group (26.8 [47.7] mg/dL) compared to those in the *ACVRL1* group (39.9 [86.4] mg/dL, *p* = 0.024). The mean hemoglobin levels were 14.1 ± 2.0 g/dL, with no significant differences between groups (Table S3). The renal function parameters (Table S4) also varied by genotype, with higher serum creatinine levels in the *ENG* group (0.85 ± 0.14 mg/dL) than those in the *ACVRL1* group (0.79 ± 0.13 mg/dL, *p* = 0.035). Similarly, the glomerular filtration rates were lower in the *ENG* group when estimated using both MDRD4 (83.7 ± 9.9 vs. 90.3 ± 12.3 mL/min/1.73 m^2^, *p* = 0.008) and CKD-EPI (92.7 ± 11.7 vs. 97.8 ± 11.0 mL/min/1.73 m^2^, *p* = 0.042). Regarding liver function (Table S5), the AST, ALT, and GGT levels were significantly higher in the *ACVRL1* group, with *p*-values of 0.016, 0.030, and 0.053, respectively. The lipid profiles (Table S6) showed higher triglyceride levels in the *ENG* group (74.5 mg/dL [49.0]) compared to those in the *ACVRL1* group (57.5 mg/dL [41.0], *p* = 0.006), while total cholesterol, LDL, and HDL showed no significant differences.

### 3.7. Mutational Variants and Their Implications

**Figure 2:**
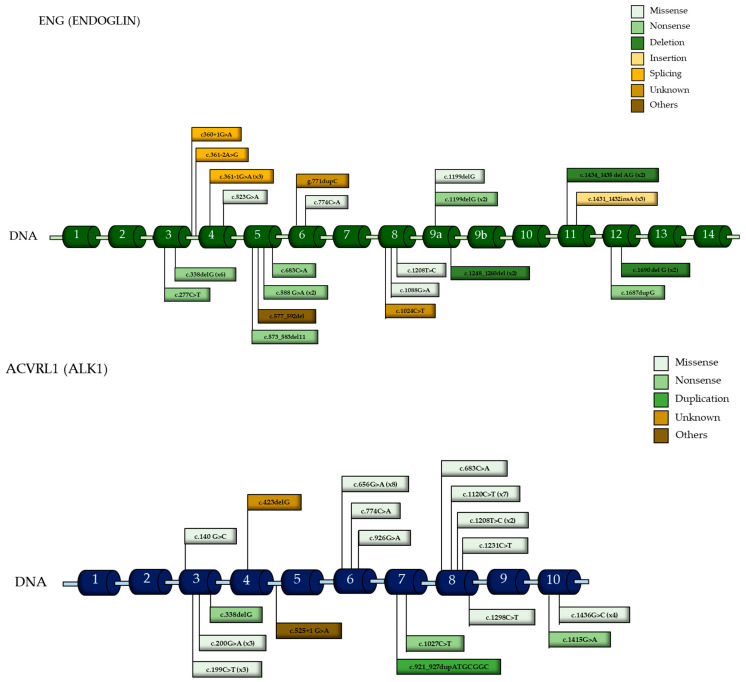
A genomic map of the different pathogenic variants identified.

The most frequently affected exons in the *ENG* gene were exons 3 and 9, affected in 16.3% of patients, followed by exons 4 and 5, which were altered in 9.3%. In the *ACVRL1* gene, the most frequently mutated exon was exon 8, observed in 28.6% of cases, followed by exon 6 (23.8%) and exon 3 (19%). Mutations in non-coding regions were rare, found in only 7% of patients with ENG mutations and 2.4% of those with *ACVRL1* mutations (Figure 2).

**Figure 3:**
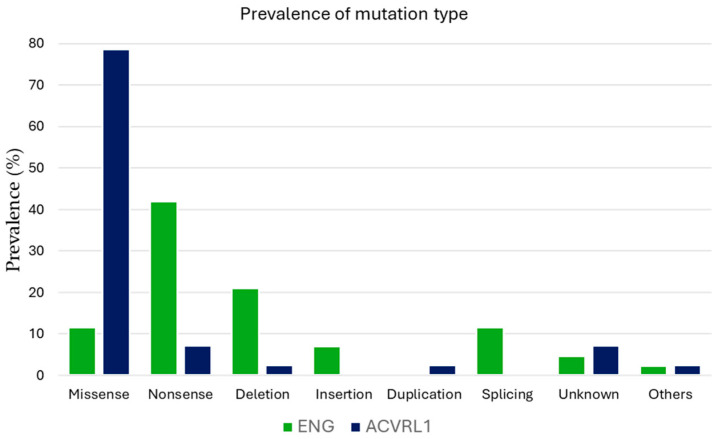
The distribution of pathogenic variants per each phenotype.

The most frequent type of mutation in the *ENG* gene was nonsense, appearing in 41.9% of patients, followed by deletions (20.9%), and missense and splicing mutations (11.6% each). In contrast, missense mutations were predominant in the *ACVRL1* gene, occurring in 78.6% of patients (Figure 3 and Table S1). Truncating mutations were significantly more common in the *ENG* group (69.8% vs. 14.3%). The logistic regression analysis confirmed truncating variants as a significant predictor of anemia, along with sex and age, while *ENG* genotype alone was not an independent risk factor (Table S2). However, the same analysis was performed between epistaxis severity and mutation type with no significant findings.

### 3.8. Cardiovascular Risk

Using the SCORE2 scale, the 10-year cardiovascular risk was 2.4% ± 2.4, consistent with low to moderate risk, with no significant differences between genotypes (Table S7).

### 3.9. Quality of Life and Phenotype Correlations

**Figure 4:**
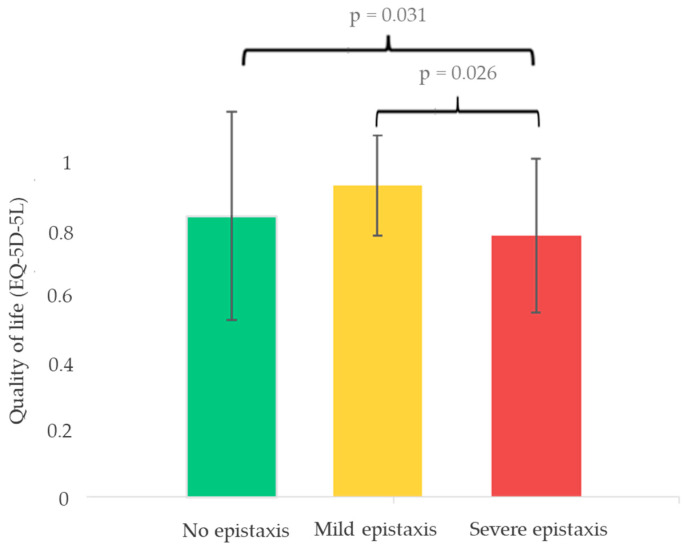
The quality of life assessment (EQ-5D-5L) according to the amount of epistaxis.

**Figure 5:**
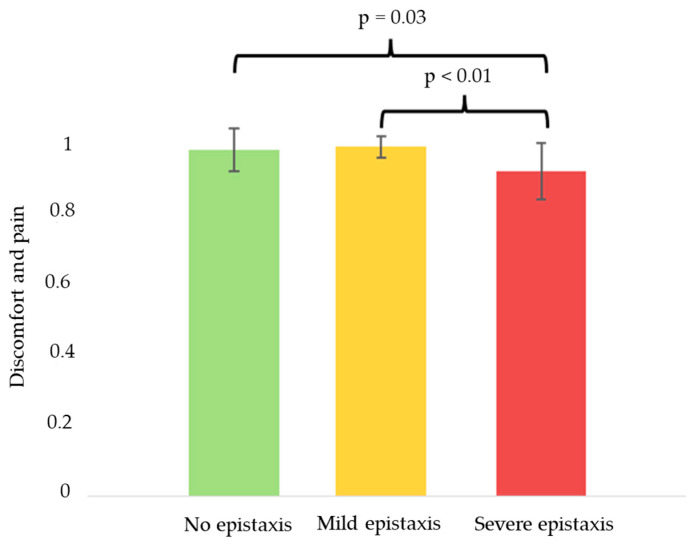
The discomfort and pain component of the EQ-5D-5L according to the amount of epistaxis.

No significant differences were observed between the different components of the EQ-5D-5L scale and the genotype of the patients (Table S8). However, patients with more severe phenotypes, characterized by greater epistaxis and anemia, had significantly worse quality of life scores, with *p*-values of 0.031 and 0.026, respectively (Figure 4 and Table S9). Dividing the quality of life scale by each of its components, it was identified that the only significant one was the sphere of pain/discomfort (Figure 5).

## 4. Discussion

This study explores the genotype–phenotype relationship in HHT, expanding on previous research by analyzing the clinical manifestations, laboratory parameters, cardiovascular risk, and quality of life in a Spanish cohort managed at a reference center for this condition.

Clinically, this study confirms differences in phenotypic expressivity between the *ENG* and *ACVRL1* variants, consistent with prior findings [10,11]. Patients with the pathogenic *ENG* variant developed epistaxis at younger ages than those with *ACVRL1* mutations, a trend previously reported by Lesca et al. [11]. Moreover, when analyzing individual components of the Epistaxis Severity Score (ESS), it was observed that patients with *ACVRL1* mutations had longer durations of epistaxis compared to those with *ENG* mutations. This aligns with prior studies [11] which have suggested that while the onset of epistaxis is later in HHT2, the severity may be greater in this group. Supporting this hypothesis, our biochemical analysis revealed that patients with *ACVRL1* mutations exhibited lower iron levels and transferrin saturation compared to these values in those with *ENG* mutations. However, paradoxically, the ferritin levels were higher in the *ACVRL1* group. This discrepancy may be explained by additional factors such as iron supplementation or underlying pro-inflammatory states [12]. These findings corroborate the existing literature, reinforcing the concept that although one genotype presents with an earlier and more expressive clinical phenotype, both genotypes can lead to severe complications, particularly anemia [4]. These findings are clinically relevant as they may help with prioritizing clinical follow-up, allowing for better care for epistaxis and its consequences in patients with HHT2 in order to avoid increased risks or a worsening quality of life in these patients due to increased epistaxis severity.

Despite the greater severity of epistaxis and iron deficiency in HHT2, the higher prevalence of pulmonary AVMs in HHT1 is a critical factor influencing overall survival. Pulmonary AVMs are known to contribute to life-threatening complications, including paradoxical embolism, brain abscesses, and severe hypoxemia, which may ultimately have a greater impact on mortality than chronic anemia [13,14]. This could explain the observed discrepancy between the mortality rates and the apparent severity of the phenotypic expression in the HHT2 patients. While anemia and recurrent epistaxis significantly impact quality of life [3], pulmonary AVMs in HHT1 are more likely to determine long-term survival [15], emphasizing the importance of early screening and intervention in this group.

Regarding AVMs in the internal organs, our study demonstrates a clear predominance of pulmonary and cerebral AVMs in the *ENG* patients, while hepatic AVMs were more frequent in the *ACVRL1* group. These results are consistent with prior research highlighting genotype-dependent organ involvement patterns [10]. The pathophysiological basis for this discrepancy remains an active area of investigation, with recent studies suggesting the differential roles of *ENG* and *ACVRL1* in vascular homeostasis and angiogenesis [16]. Notably, the early diagnosis of CNS AVMs in *ENG* patients underscores the importance of routine screening in this subgroup, given the potential for life-threatening complications [17].

The analysis of the family history data revealed that most HHT-related deaths occurred in patients with *ENG* mutations. Although statistical significance was not reached, this trend suggests a more severe clinical course in *ENG* patients. Causes of death included hemorrhagic stroke, severe anemia, and massive epistaxis, highlighting the need for proactive management strategies in this group.

This work also considered the main biochemical variables and identified that HHT1 patients have significantly lower glomerular filtration rates than HHT2 patients. This phenomenon may be explained by the higher number of pulmonary AVMs in these patients. Indeed, patients with pulmonary AVMs are more likely to undergo chest CT angiography with iodinated contrast agents and then pulmonary arteriography due to embolization. This finding may call into question the need to be more conservative with these patients, especially if pulmonary AVMs are identified at an early age. Less invasive follow-up tests such as bubble echocardiograms could be performed to space out the contrast tests [18].

An important aspect of this study was the evaluation of cardiovascular risk using the SCORE-2 model. The majority of the patients were classified as having a low to moderate 10-year cardiovascular risk. This suggests that when assessed using the conventional risk models, HHT patients do not appear to have an increased likelihood of cardiovascular events. However, patients with *ACVRL1* mutations had higher triglyceride levels, albeit within the normal limits. Additionally, *ACVRL1* has been implicated in lipid metabolism and LDL particle mobilization [19]. It remains unclear whether the inflammatory burden associated with HHT influences cardiovascular risk, a phenomenon well documented in other systemic diseases such as systemic lupus erythematosus [20]. Longitudinal studies with extended follow-ups are needed to determine whether the predicted cardiovascular risk in HHT patients aligns with actual event rates. If discrepancies arise, an HHT-specific cardiovascular risk stratification model may be necessary.

From a quality of life perspective, the initial analysis using the EQ-5D-5L scale did not reveal significant differences between genotypes. However, when stratified by phenotype, it became evident that patients with anemia and more severe epistaxis had significantly lower quality of life scores. These findings are in line with previous studies emphasizing the impact of chronic bleeding on daily functioning [3]. Notably, when analyzing the individual EQ-5D-5L domains, “discomfort” emerged as the most affected component. This may reflect the broad nature of the question, which resonated more with the patients compared to other domains that are tailored to different pathologies. These results underscore the need for disease-specific quality of life measures to optimize patient follow-up and treatment assessments [21]. It should also be a priority to implement these scales in HHT practices in order to guide the treatment towards the clinical aspects that affect patients the most.

Beyond the distinction between *ENG* and *ACVRL1* mutations, this study highlights the importance of identifying the specific type of mutation, as truncating versus non-truncating variants may play a crucial role in phenotype development and prognosis [11]. Our findings suggest that truncating mutations are significantly associated with anemia, regardless of the affected gene. The same findings were not evident in relation to epistaxis. This may be partly due to the evaluation of the severity of epistaxis through the ESS, which is dependent on the patient’s perception. However, it may be that the phenotype of the patients is not entirely explained by the genotype but by other factors (e.g., environmental). This supports growing recognition that the functional consequences of genetic alterations, rather than solely the mutated gene, may determine disease severity. This leads to the importance of a comprehensive genetic study, as it could help to identify patients who may possibly have a worse phenotype. Future studies should explore the implications of mutation type for clinical outcomes further, as this information could refine risk stratification and therapeutic decision-making.

This study has several limitations. First, its cross-sectional design does not allow for an assessment of longitudinal disease progression or the causality between genotype and phenotype. A prospective study with a long-term follow-up would be necessary to understand the natural history of HHT manifestations better. Second, the sample size is relatively small, limiting the statistical power for some analyses, particularly regarding rare complications such as CNS VMs. Multicenter studies with larger cohorts would improve the generalizability. Third, potential selection bias exists, as the patients were recruited from a specialized center, which may have over-represented more severe cases or those actively seeking treatment. In the same way, being a nested study in a prospective research project, the inclusion and exclusion criteria may have led to biases, such as the exclusion of patients with recent cardiovascular events. Lastly, despite efforts to standardize the data collection, variability in medical records and patient-reported outcomes may have introduced information bias. Future research should aim to validate these findings in independent populations and incorporate objective biomarkers to refine the genotype–phenotype correlations.

## 5. Conclusions

Overall, this study reinforces the importance of a genotype-driven approach to HHT management. While *ENG* patients require vigilant screening for pulmonary AVMs, *ACVRL1* patients may benefit from close monitoring of epistaxis severity, anemia, and lipid metabolism. Furthermore, the recognition that mutation type may influence phenotype severity suggests that the genetic classification should extend beyond affected genes to include the mutation characteristics. Genetical tests should be a priority in the evaluation of HHT patients to characterize mutation types and their clinical manifestations further. More studies should be conducted using quality of life scales adapted to HHT, and prospective studies including cardiovascular events should be conducted in order to adapt the risk tools to the disease. However, clinicians should prioritize the performance of quality of life scales in these patients in order to assess the real impact of their treatments and prioritize the therapies used in each patient. Future research should focus on validating these findings in larger cohorts and exploring novel therapeutic strategies for mitigating the genotype-specific complications of HHT.

## Figures and Tables

**Table 1:** General characteristics of the patients.

	Total (n = 85)	ENG (HHT1) (n = 43)	ACVRL1 (HHT2) (n = 42)	ORs * [IC95%]	*p*
Age, years				-	0.352
Mean ± SD	48.6 ± 14.3	47.2 ± 14.4	50.1 ± 14.3		
Range (min–max)	20–76	20–74	22–76		
Women, n (%)	50 (58.8%)	25 (58.1%)	25 (59.5%)	0.94 [0.39 to 2.24]	0.897
Family history, n (%)					
Relatives affected by HHT	78 (91.8%)	38 (88.4%)	40 (95.2%)	0.38 [0.06 to 2.07]	0.250
Brother/sister	48 (56.5%)	20 (46.5%)	28 (66.7%)	0.43 [0.18 to 1.04]	0.061
Son/daughter	28 (32.9%)	16 (37.2%)	12 (28.6%)	1.48 [0.59 to 3.68]	0.397
Father/mother	66 (77.6%)	31 (72.1%)	35 (83.3%)	0.51 [0.18 to 1.47]	0.214
Grandfather/mother	27 (31.2%)	12 (27.9%)	15 (35.7%)	0.69 [0.27 to 1.74]	0.440
Grandson/daughter	2 (2.4%)	1 (2.3%)	1 (2.4%)	0.97 [0.05 to 16.1]	0.987
Deceased relatives related to the disease	24 (28.2%)	16 (18.8%)	8 (9.4%)	2.51 [0.93 to 6.76]	0.063
Causes of death of relatives					
Digestive bleeding	10 (11.8%)	6 (14.0%)	4 (9.5%)	1.54 [0.40 to 5.9]	0.526
Hemorrhagic stroke	7 (8.2%)	6 (14.0%)	1 (2.4%)	6.64 [0.76 to 57.8]	0.052 *
Brain abscess	0 (0.0%)	0 (0.0%)	0 (0.0%)	-	-
Other ^1^	11 (12.9%)	7 (16.7%)	4 (9.5%)	1.84 [0.49 to 6.84]	0.354

* ORs: odds ratios for ENG vs. ACVRL1 in the univariate analysis. ^1^ Other causes of HHT-related familial mortality: anemia (3 cases), massive epistaxis (2 cases), pulmonary hemorrhage (2 cases), portal hypertension (1 case), HOHF (1 case), 2 unspecified. HOHF: high-output heart failure.

**Table 2:** Characteristics of epistaxis and treatment received.

	Total (n = 85)	ENG (HHT1) (n = 43)	ACVRL1 (HHT2) (n = 42)	ORs * [IC95%]	*p*
Epistaxis	81 (95.3%)	42 (97.7%)	39 (92.9%)	3.23 [0.32 to 32.3]	0.294
Clinical symptoms related to epistaxis, n (%)					
ESS	2.49 ± 1.79	2.40 ± 1.66	2.68 ± 2.08	-	0.645
Severity of epistaxis					0.142
Severe (ESS ≥ 4)	13 (15.3%)	4 (9.3%)	9 (21.4%)	0.376	
Mild/none (ESS < 4)	72 (84.7%)	39 (90.7%)	33 (78.6%)	[0.106 to 1.333]	
Frequency of epistaxis				-	0.531
At least once a day	21 (24.7%)	10 (23.3%)	11 (26.2%)		
At least once a week	39 (45.9%)	18 (41.9%)	21 (50.0%)		
At least once a month	25 (29.4%)	15 (34.9%)	10 (23.8%)		
Duration of epistaxis				-	0.006
More than 5 min	16 (18.8%)	7 (16.3%)	9 (21.4%)		
1–5 min	41 (48.2%)	15 (34.9%)	26 (61.9%)		
Less than 1 min	28 (32.9%)	21 (48.8%)	7 (16.7%)		
Intensity of epistaxis				0.676 [0.258 to 1.772]	0.425
Sheets of blood	23 (27.1%)	10 (23.3%)	13 (31.0%)		
Drips of blood	62 (72.9%)	33 (76.7%)	29 (69.0%)		
Needs to visit the emergency room due to epistaxis	3 (3.5%)	2 (4.7%)	1 (2.4%)	2000 [0.174 to 22.927]	1.000
Currently anemic	10 (11.8%)	5 (11.6%)	5 (11.9%)	0.974 [0.260 to 3.644]	0.968
Needs transfusions	1 (1.2%)	0 (0.0%)	1 (2.4%)	-	0.494

* ORs: odds ratios for ENG vs. ACVRL1 in the univariate analysis.

**Table 3:** Location of arteriovenous malformations.

	Total (n = 85)	ENG (HHT1) (n = 43)	ACVRL1 (HHT2) (n = 42)	ORs * [IC95%]	*p*
Internal organ involvement, n (%)					
Pulmonary AVMs	29 (34.1%)	25 (58.1%)	4 (9.5%)	13.19 [3.9 to 43.5]	<0.001
Age at diagnosis of pulmonary AVMs, years, median [IQR]	39.0 [24.0]	39.0 [25.0]	41.5 [20.3]	-	0.635
CNS VMs	12 (14.1%)	9 (20.9%)	3 (7.1%)	3.44 [0.86 to 3.75]	0.068
Brain VMs	11 (12.9%)	8 (18.6%)	3 (7.1%)	2.97 [0.7 to 12.08]	0.115
Spinal VMs	1 (1.2%)	1 (2.3%)	0 (0.0%)	-	0.320
Age at diagnosis of CNS VMs, years, median [IQR]	28.0 [10.0]	26.0 [8.0]	32.0 [6.0]	-	0.630
Hepatic VMs	15 (17.6%)	5 (11.6%)	10 (23.8%)	0.42 [0.13 to 1.35]	0.141
Age at diagnosis of hepatic VMs, years, median [IQR]	48.5 [19.5]	41.0 [24.0]	49.0 [17.8]	-	0.304
Digestive VMs	10 (11.8%)	4 (9.3%)	6 (14.3%)	0.61 [0.16 to 2.35]	0.476
Age at diagnosis of digestive VMs, years, median [IQR]	57.5 [19.3]	56.5 [13.0]	58.0 [29.0]	-	0.610
All types of VMs	44 (51.8%)	27 (62.8%)	17 (40.5%)	2.48 [1.03 to 5.94]	0.040
Age at diagnosis of VMs, years, median [IQR]	39.0 [25.0]	37.0 [26.0]	41.0 [22.0]	-	0.198

* ORs: odds ratios for ENG vs. ACVRL1 in the univariate analysis. VMs: vascular malformations; AVMs: arteriovenous malformations; IQR: interquartile range.

## Data Availability

The data involved in conducting this study are available upon request from the corresponding author.

## Supplementary Materials

The following supporting information can be downloaded at https://www.mdpi.com/article/10.3390/jcm14134409/s1. Table S1: Types of pathogenic variants; Table S2: Logistic regression analysis of anemia in relation to truncated variants adjusted for confounding variables (age, sex and genetics); Table S3: Erythropoiesis data; Table S4: Kidney function; Table S5: Liver function values in patients with HHT; Table S6: Description of lipid profile values in patients with HHT; Table S7: Cardiovascular risk; Table S8: Description of the components of the EQ-5D-5L according to genotype; Table S9: Quality of life, according to the EQ-5D-5L scale, by severity of the disease.

## Abbreviations

The following abbreviations are used in this manuscript:

HHT	Hemorrhagic Hereditary Telangiectasia
ESS	Epistaxis Severity Score
GFR	Glomerular Filtration Rate
VM	Vascular Malformation
AVM	Arteriovenous Malformation
CNS	Central Nervous System
