# Supplementary material for: Genotype–Phenotype Relationship in Hereditary Hemorrhagic Telangiectasia: Quality of Life and Cardiovascular Risk Evaluation
Source: J Clin Med. 2025 Jun 20;14(13):4409. doi: 10.3390/jcm14134409 (PMC12249744; doi:10.3390/jcm14134409)
Supplement: Supplementary file 1 [file jcm-14-04409-s001.zip › jcm-3665315-supplementary.pdf]

Journal of
Clinical Medicine

MDPI

Supplemental Material:

Supplementary Table S1. Types of pathogenic variants

Type of pathogenic variant	Population %	*ENG* (%)	*ACVRL1* (%)
Missense	38 (44.7%)	5 (11.6%)	33 (78.6%)
Nonsense	21 (24.7%)	18 (41.9%)	3 (7.1%)
Deletion	10 (11.8%)	9 (20.9%)	1 (2.4%)
Insertion	3 (3.5%)	3 (7.0%)	0 (0.0%)
Duplication	1 (1.2%)	0 (0.0%)	1 (2.4%)
Splicing	5 (5.9%)	5 (11.6%)	0 (0.0%)
Others	2 (2.4%)	1 (2.3%)	1 (2.4%)
Unknown	5 (5.9%)	2 (4.7%)	3 (7.1%)
Total	85 (100%)	43 (100%)	42 (100%)

Supplementary Table S2. Logistic regression analysis of anemia in relation to truncated variants adjusted for confounding variables (age, sex and genetics)

Variable	B	Standard error	p-value	Exp (B)	95% CI for Exp (B)
Intercept	3,933	1,129	0.000*	-	-
Truncated	1,554	0.664	0.019*	4,731	[1,287, 17,385]
Sex	-1,560	0.566	0.006*	0.210	[0.069, 0.636]
Age	-0.064	0.020	0.002*	0.938	[0.902, 0.977]
ENG	-0.547	0.633	0.388	0.579	[0.167, 2.001]

B: Estimated coefficient.

Standard error: Standard error of the coefficient.

Sig.: p value of the Wald test. Values less than 0.05 are considered significant (*).

Exp (B): Odds ratio, which represents the ratio of probabilities for each predictor variable.

95% CI for Exp (B): 95% confidence interval for Exp (B). If the interval does not include 1, the effect is considered statistically significant.

Supplementary Table S3. Erythropoiesis data

	**Total (n=85)**	***ENG* (HHT1) (n=43)**	***ACVRL1* (HHT2) (n=42)**	**p**
Hemogram parameters				
Hemoglobin (g/dL)	14.1 ± 2.0	14.3 ± 2.0	13.9 ± 2.1	0.329
Mean corpuscular volume (fl)	91.7 ± 7.0	91.7 ± 7.2	91.8 ± 6.9	0.949
Hematocrit (%)	43.2 ± 5.4	43.8 ± 5.4	42.6 ± 5.4	0.294
Biochemical parameters				
Iron (ug /dL)	79.4 ± 56.6	92.3 ± 72.7	66.8 ± 30.5	0.043
Transferrin saturation (%)	24.1 ± 19.9	28.7 ± 26.2	19.7 ± 9.2	0.042
Transferrin (mg/ dL)	283.5 ± 42.9	291.1 ± 42.5	276.3 ± 42.3	0.120
Ferritin (mg/dL), median [IQR]	34.6 [62.3]	26.8 [47.7]	39.9 [86.4]	0.024

IQR: Interquartile range

Supplementary Table S4. Kidney function

	**Total** (n=85)	**ENG (HHT1)** (n=43)	**ACVRL1 (HHT2)** (n=42)	**p**
Creatinine (mg/ dL)	0.82 ± 0.14	0.85 ± 0.14	0.79 ± 0.13	0.035
Glomerular filtration rate (MDR4) mL /min/1.73m^2	87.0 ± 11.6	83.7 ± 9.9	90.3 ± 12.3	0.008
Glomerular filtration rate (CKD-EPI) mL /min/1.73m^2	95.2 ± 11.6	92.7 ± 11.7	97.8 ± 11.0	0.042
Urea (mg/ dL)	31.5 ± 8.7	31.4 ± 8.6	31.7 ± 9.0	0.852
Uric Acid (mg/ dL)	5.2 ± 1.3	5.2 ± 1.2	5.1 ± 1.5	0.587
Sodium (mmol/L)	139.6 ± 2.1	139.4 ± 2.3	139.7 ± 1.9	0.626
Chlorine (mmol/L)	106.2 ± 2.2	106.4 ± 2.3	106.0 ± 2.1	0.316
Potassium (mmol/L)	4.2 ± 0.3	4.3 ± 0.3	4.2 ± 0.3	0.322

Supplementary Table S5. Liver function values in patients with HHT

	**Total** (n=85)	**ENG (HHT1)** (n=43)	**ACVRL1 (HHT2)** (n=42)	**p**
Total proteins (mg/ dL)	7.0 ± 0.6	7.1 ± 0.5	6.9 ± 0.6	0.113
Total bilirubin (mg/ dL), median [IQR]	0.65 [0.56]	0.58 [0.48]	0.73 [0.77]	0.373
AST (mg/ dL), median [IQR]	22.0 [10.0]	20.0 [8.0]	23.5 [10.0]	0.016
ALT (mg/ dL), median [IQR]	18.0 [16.0]	17.0 [12.0]	24.0 [17.0]	0.030
GGT (mg/ dL), median [IQR]	21.0 [25.0]	19.0 [13.0]	25.0 [34.0]	0.053
FA (mg/ dL), median [IQR]	66.5 [26.0]	66.0 [17.0]	67.0 [30.0]	0.785
LDH (mg/ dL), median [IQR]	190.5 [54.0]	193.0 [56.0]	190.0 [49.0]	0.398

IQR: Interquartile range

Supplementary Table S6. Description of lipid profile values in patients with HHT

	**Total (n=85)**	**ENG (HHT1) (n=43)**	**ACVRL1 (HHT2) (n=42)**	**p**
Biochemical parameters				
Total cholesterol (mg/ dL)	188.5 ± 34.0	190.9 ± 32.9	186.0 ± 35.4	0.510
LDL cholesterol (mg/ dL)	113.0 ± 29.6	113.8 ± 30.2	112.1 ± 29.2	0.794
HDL cholesterol (mg/ dL)	58.6 ± 15.5	58.5 ± 15.2	58.8 ± 16.0	0.939
Triglycerides (mg/ dL), median [IQR]	65.0 [49.0]	74.5 [61.0]	57.5 [41.0]	0.006

LDL: Low-density lipoprotein; HDL: High-density lipoprotein; IQR: Interquartile range.

Supplementary Table S7. Cardiovascular risk

Cardiovascular Risk (SCORE2)				
	**Total (n=85)**	***ENG* (HHT1) (n=43)**	***ACVRL1* (HHT2) (n=42)**	**p**
10-year CV risk (%), Mean ± SD<br>Range (Min.- Max)	(n=79)<br>2.4 ± 2.4<br>0.1 – 11.8	(n=38)<br>2.2 ± 2.1<br>0.1 – 9.0	(n=41)<br>2.7 ± 2.6<br>0.2 – 11.8	0.404
Age-adjusted CV risk group<br>Low<br>Intermediate<br>High	 67 (84.8%)<br>12 (15.2%)<br>0 (0.0%)	 32 (84.2%)<br>6 (15.8%)<br>0 (0.0%)	 35 (85.4%)<br>6 (14.6%)<br>0 (0.0%)	0.886

CV: Cardiovascular

Supplemental Table S8. Description of the components of the EQ-5D-5L according to genotype

**Quality of life (EQ-5D-5L), mean ± SD**	**Total** (n=85)	**ENG (HHT1)** (n=43)	**ACVRL1 (HHT2)** (n=42)	**p-value**
Mobility	0.97 ± 0.08	0.96 ± 0.09	0.99 ± 0.05	0.160
Self-care	0.99 ± 0.04	0.98 ± 0.05	0.99 ± 0.03	0.272
Activities of daily living	0.99 ± 0.04	0.99 ± 0.04	0.99 ± 0.04	1,000
Discomfort or pain	0.97 ± 0.05	0.98 ± 0.05	0.97 ± 0.06	0.737
Anxiety or depression	0.96 ± 0.07	0.96 ± 0.07	0.97 ± 0.06	0.564
Total quality of life	0.89 ± 0.21	0.87 ± 0.21	0.91 ± 0.20	0.407

Supplementary Table S9. Quality of life, according to the EQ-5D-5L scale, by severity of the disease

		**Quality of life (EQ-5D-5L), mean ± SD**	**p-value**
Severity of Epistaxis	No epistaxis	0.84 ± 0.31	0.031
	Mild nosebleed	0.93 ± 0.15	
	Moderate nosebleed	0.78 ± 0.23	
Anemia or IV iron	No	0.94 ± 0.17	0.026
	Yes	0.84 ± 0.22	

IV: Intravenous
